# Translation and Validation of the Malay Version of the WHO-5 Well-Being Index: Reliability and Validity Evidence from a Sample of Type 2 Diabetes Mellitus Patients

**DOI:** 10.3390/ijerph19074415

**Published:** 2022-04-06

**Authors:** Aida Farhana Suhaimi, Shahidah Mohamed Makki, Kit-Aun Tan, Umi Adzlin Silim, Normala Ibrahim

**Affiliations:** 1 Department of Psychiatry, Faculty of Medicine and Health Sciences, Universiti Putra Malaysia, Serdang 43400, Malaysia; normala_ib@upm.edu.my; 2 Department of Psychiatry and Mental Health, Hospital Putrajaya, Ministry of Health, Putrajaya 62250, Malaysia; 3 Hospital Bahagia Ulu Kinta, Ministry of Health, Tanjung Rambutan 31250, Malaysia; shahidahmakki@gmail.com; 4 Department of Psychiatry and Mental Health, Hospital Serdang, Ministry of Health, Kajang 43000, Malaysia; umiadzlin@gmail.com

**Keywords:** WHO-5, well-being, factor structure, reliability, validity, diabetes, Malaysia, primary care

## Abstract

The translation and validation process of the WHO-5 Well-Being Index (WHO-5) into Malay is still not yet available. This study is the first psychometric evaluation of the Malay version of the WHO-5 in a sample of 127 primary care patients with type 2 diabetes mellitus. We evaluated the internal consistency and 5-week test–retest reliability of the WHO-5 Malay, and three aspects of its validity—first, the factorial validity in relation to the factor structure of the WHO-5 Malay; second, the concurrent validity in relation to depression and diabetes-related distress; and third, the convergent validity in relation to diabetes management self-efficacy and diabetes self-care behaviors. This study had two phases. Phase 1 involved the translation of the WHO-5 into Malay language following established procedures, whereas Phase 2 involved the validation of the WHO-5 Malay. Excellent internal consistency and 5-week test–retest reliability estimates were obtained. The factorial validity of the WHO-5 was found to be unidimensional. As for concurrent validity, the WHO-5 Malay was found to be negatively correlated with depression and diabetes-related distress. The WHO-5 was found to be correlated with diabetes management self-efficacy and diabetes self-care behaviors, thereby establishing convergent validity. The WHO-5 Malay has reliable and valid psychometric properties and represents a promising tool that informs healthcare providers in making effective and holistic diabetes management.

## 1. Introduction

Despite advancement in the clinical management of diabetes, prevalence of such chronic disease continues to rise [1]. Indeed, many patients struggle to cope living with diabetes and experience negative psychosocial consequences [2]. In a world-wide study involving participants from 13 countries, it was reported that 41% of the participants with diabetes had poor psychological well-being [as measured by the WHO-5 Well-Being Index; [3]. In Malaysia, the prevalence of elevated depressive symptoms in diabetes patients was found to be ranging from 11.5% to 41.7%, and 49.2% of them experiencing diabetes-related distress [4,5,6].

Mental health issues have been consistently found to hold significant barriers to effective management [7], patient engagement to healthcare use [8], and diabetes control [9]. This is even more so in Malaysia [4,5,6]. In Malaysia or elsewhere, it appears that diabetes management has mainly focused on medical management, and very little effort has been made to address psychosocial health and overall well-being [10,11]. There is an urgent call for comprehensive diabetes management that includes psychosocial aspects of care [12]. Psychosocial care and self-management vary between Types 1 and 2 diabetes in that Type 2 diabetes patients significantly benefitting from diabetes management that addresses the psychosocial aspect of the disease [13]. Managing the psychosocial aspect of the disease in Type 2 diabetes patients has shown evidence in improving glycated hemoglobin levels, dietary behavior, and quality of life [13] and in reducing depressive and anxiety symptoms [14]. Hence, assessment of, and management for, diabetes can no longer just solely rely on the presentation of the disease [15], but also requires a broader understanding of its impact on individuals’ psychological well-being. To this end, assessing the well-being and psychosocial aspect of management in Type 2 diabetes patients would be of great clinical value.

### 1.1. The WHO-5 Well-Being Index

Integration of psychosocial care with usual medical management has consistently been established as the way towards effective management [16,17,18]. Therefore, as part of the diabetes management, assessing psychological well-being of patients is utmost important, especially within the primary care setting where the bulk of patients with diabetes and possible mental health issues are initially presented [17,19]. There are several approaches in defining and assessing well-being, and several tools have been developed over the years to measure well-being. Such approaches have resulted in a wide array of conceptualizations, with some authors focusing on subjective well-being (e.g., the Unity Index of Psychological Well-being), others on life satisfaction (e.g., the Life Satisfaction Research Questionnaire), and still others on quality of life (e.g., the WHO-Quality of Life Scale) and wellness (e.g., the Five Factor Wellness Evaluation of Lifestyle) [20]. Variabilities also exist within a wide range of well-being instruments with some authors assessing the positive characteristics of well-being or absence of poor well-being (e.g., the EuroQOL-EQ-5D), whereas others incorporate a multidimensional approach (e.g., the Ryff Scales of Psychological Well-Being) [19]. In areas of diabetes, assessments of well-being have been developed to measure negative affect or lack of mental health (e.g., the Short-Form 36 Mental Health sub-scale) or the presence of mental disorders such as depression (e.g., the Patient Health Questionnaire; [21,22]). Rather than a measurement on the presence of mental illness or poor mental health, the WHO-5 Well-Being Index (WHO-5) is designed to assess positive functioning or psychological well-being [21].

The WHO-5 has been widely used in studies related to endocrinology, specifically within the areas of diabetes [23]. Based on a review on well-being measurement, the WHO-5 was found to be the sole well-being measurement that is not only brief, but also has good psychometric properties [19]. Thus, making it an invaluable tool within the busy primary healthcare setting where the bulk of patients are presented with diabetes and mental health issues. With its five positively worded items, the WHO-5 provides a measurement of well-being that can be monitored over time in the management of diabetes [24]. Participants take about 2 min to complete the scale. In its initial construction, there are 28 items assessing depression, distress, anxiety, as well as general health and well-being [21]. These items were inclusive of both the positive and negative aspects of well-being. With further analyses, the 28-item scale was further reduced to 10 items and later to five items, eliminating negative aspects of well-being and narrowing its focus on the positive aspects of well-being [25]. Though brief, the WHO-5 has been shown to be a highly preferable well-being scale within the primary health care [24]. According to a systematic review on the psychometric properties of the WHO-5, the scale has demonstrated evidence for validity, sensitivity, and specificity. Previous studies have consistently reported a unidimensional WHO-5 model [26,27,28]. The WHO-5 was found to be a reliable and valid scale for not only screening depression, but also assessing psychological outcome (see [23] for a review).

### 1.2. The Present Study and Hypotheses

Since its first development in 1998, the WHO-5 has been translated into more than 30 languages (e.g., Thai, Japanese, Portuguese, Arab, and Chinese) and is widely used in different population groups across different fields (e.g., endocrinology, psychiatry, psychology, geriatrics, neurology, cardiology, oncology, and pain; [23]). Despite its promising potential as a health outcome measurement in both research and clinical settings [23], the WHO-5 have yet undergone a thorough translation process into the Malay language which is the national and formal language in Malaysia. Given the high local prevalence of mental health issues experienced within the diabetes population [4,5,6], availability of a readily translated brief mental health screening tool and a psychological well-being measurement would be of great value especially within the busy primary care setting in Malaysia.

Henceforth, the primary objective of the present study was to examine the reliability and validity of the Malay version of the WHO-5 Well-Being Index (WHO-5 Malay) in a sample of Type 2 diabetes mellitus (T2DM) patients in a primary care setting. First, we hypothesized that the WHO-5 Malay would have a good internal and 5-week test–retest reliability that coincide with the routine of local diabetes clinic visits in the primary care clinics. Second, as with existing studies (e.g., [26,27,28]), we hypothesized that the WHO-5 Malay would conform to the one-factor structure (i.e., unidimensional) of the original WHO-5. Third, based on the existing literature, we hypothesized that WHO-5 Malay would be negatively correlated with concurrent measures of depression (i.e., the Malay version of the Patient Health Questionaire-9; [26,28]) and emotional problems (i.e., the Malay version of the Problem Areas in Diabetes; [26]) and positively correlated with convergent measures of diabetes management self-efficacy (i.e., the Malay version of the Diabetes Management Self-Efficacy Scale; [29]) and diabetes self-care behaviors (i.e., the Malay version of the Summary of Diabetes Self-Care Activities Measure; [30]).

## 2. Materials and Methods

### 2.1. Participants

This manuscript was part of an intervention study examining the effectiveness of a well-being self-management program in T2DM patients in primary care clinics [31]. We used subject-to-item ratios of 20:1 to calculate sample size [32]. The sample size requirement for a psychometric validation on a 5-item scale such as the WHO-5 Malay would be at least 100 participants. To this end, 127 participants were recruited. Of these, only 119 participants responded and completed reassessment at 5-week follow-up with an attrition rate of 6.7%. The participants’ ages ranged from 25 to 63 years (50.4% male and 49.6% female) with a mean of 47.1 years (*SD* = 8.62). The present sample were predominately Malay (93.7%), Islam (95.3%), married (92.15%), employed full time (78.0%), and had attained secondary level education (36.2%). As far as diabetes complications are concerned, 79.4% of the participants reported no diabetes complications. The average number of years that the patients had suffered from diabetes was 5.7 years (*SD* = 4.94).

### 2.2. Study Eligibility

Included participants were aged between 18 to 65 years old, had a known diagnosis of T2DM, currently attending health clinics located in the Federal Territory of Putrajaya, Malaysia, and were able to provide consent independently without assistance. Those who were illiterate, unable to converse in Malay or English, and had a serious acute medical illness (e.g., delusional, delirium) during the intake were excluded from the study.

### 2.3. Ethical and Translation Approval

Ethical approval for this study was obtained from the Medical Research and Ethics Committee, Ministry of Health Malaysia. Permission to use and translate the WHO-5 was obtained from the original scale developer.

### 2.4. Study Procedure

This study included two phases. In Phase 1, we translated the WHO-5 into Malay language following the World Health Organization’s (WHO) recommendations [33]. In Phase 2, we examined the reliability and validity of the WHO-5 in a sample of diabetes patients in the primary healthcare clinic.

#### 2.4.1. Phase 1: Translation of the WHO-5 Well-Being Index into Malay Language

Based on the WHO’s [33] recommendations concerning the process of scale translation and adaptation, we translated the WHO-5 in four steps: forward translation, expert panel back translation, pre-testing, and final version. In Step 1, after obtaining approval to translate the scale from the original scale developer, two bilingual clinical psychologists and one certified translator performed the forward translation. Then, a panel of experts consisting of bilingual mental health practitioners reviewed the forward translated version. Discrepancies between the forward translated version and original WHO-5 were resolved through panel discussion. In Step 2, the forward translated version was back translated by another two bilingual clinical psychologists. The back translated version was reviewed by the panel of experts. Discrepancies between the back translated version and the original WHO-5 were highlighted, discussed, and resolved through panel discussion. At the end of this stage, the preliminary version of the WHO-5-Malay was obtained. In Step 3, the preliminary version of the WHO-5-Malay was pre-tested and reviewed by five clinical psychologists and five T2DM patients attending a primary healthcare clinic. No concerns surrounding the understandability and appropriateness of the translated items were documented. Lastly, in Step 4, the final version of WHO-5 Malay was obtained.

#### 2.4.2. Phase 2: Validation of the Malay Version of the WHO-5 Well-Being Index

Participants completed a research questionnaire containing the final version of the WHO-5 Malay along with socio-demographic measures (age, gender, ethnicity, religion, employment status, marital status, diabetes complications, duration since diagnosed with diabetes), the Patient Health Questionnaire (PHQ-9), the Problem Areas in Diabetes (PAID-20), the Summary of Diabetes Self-Care Activities Measure (SDSCA), and the Diabetes Management Self-Efficacy scale (DMSES). At 5-week follow-up, all participants were invited to complete the WHO-5 Malay for test–retest assessment, in line with the time point assessment of the intervention study [31]. Of 127 participants invited for test–retest assessment, 119 participants completed the WHO-5 Malay at 5-week after the initial assessment was done.

### 2.5. Measures

#### 2.5.1. The Malay Version of the WHO Well-Being Index

The Malay version of the WHO Well-being Index (WHO-5 Malay) is a 5-item self-report measure of emotional well-being. Participants rated items on a 6-point Likert scale ranging from 0 (*none of the time*) to 5 (*all of the time*). The raw score that ranges from 0 (*absence of well-being*) to 25 (*maximum well-being*) are then multiplied by 4 to obtain total score.

#### 2.5.2. Depression

The Malay version of the Patient Health Questionnaire (PHQ-9) is a 9-item self-report measure of depression [34]. Participants rated items on a 4-point Likert scale ranging from 0 (*not at all*) to 3 (*nearly every day*). Possible scores range from 0 to 27. Acceptable internal consistency evidence (Cronbach’s alpha estimate = 0.70) was reported for the respective scale in patients attending primary healthcare clinic in a past study [34]. Cronbach’s alpha estimate for the Malay version of the PHQ-9 in the present study was 0.90.

#### 2.5.3. Emotional Problems

The Malay version of the Problem Areas in Diabetes (PAID-20) is a 20-item self-report measure of emotional problems in patients with diabetes [35]. Participants rated items on a 5-point Likert scale ranging from 0 (*not a problem*) to 4 (*serious problem*). Possible scores range from 0 to 80. Excellent internal consistency evidence (Cronbach’s alpha estimate = 0.92) was reported for the respective scale in T2DM patients attending a public hospital in a past study [35]. Cronbach’s alpha estimate for the Malay version of the PAID-20 in the present study was 0.97.

#### 2.5.4. Diabetes Management Self-Efficacy

The Malay version of the Diabetes Management Self-Efficacy Scale (DMSES) is a 20-item self-report measure of self-efficacy in managing specific diabetes self-care behaviors [36]. Participants rated items on an 11-point Likert scale ranging between 0 (*Not at all confident*) to 10 (*totally confident*). Possible scores range from 0 to 200. Excellent internal consistency evidence (Cronbach’s alpha estimates = 0.92) was reported for the respective scale in T2DM patients attending a public hospital in a past study [36]. Cronbach’s alpha estimate for the Malay version of the DMSES in the present study was 0.95.

#### 2.5.5. Diabetes Self-Care Behaviors

The Malay version of the Summary of Diabetes Self-Care Activities Measure (SDSCA) is a 10-item self-report measure of patient’s engagement to diabetes self-care behaviors [37]. Participants rated items on an 8-point Likert scale indicating the average number of days engaged in diabetes self-care behaviors. Possible scores range from 0 days to 7 days. Acceptable to excellent internal consistency evidence (Cronbach’s alpha estimates = 0.65 to 0.91) was reported for the respective scale in T2DM patients attending a primary care clinic in a past study [37]. Cronbach’s alpha estimate for the Malay version of the SDSCA in the present study was 0.77.

### 2.6. Data Analytic Plan

We used Statistical Package Social Sciences (SPSS) 25.0 statistical software (IBM Corp., Armonk, NY, USA) and AMOS 23.0 statistical software (IBM SPSS, Chicago, IL, USA) to perform statistical analyses. Descriptive statistics were used to present study measures (see Table 1). The normal distribution of study variables was assessed by examining skewness and kurtosis indices. Acceptable ranges for skewness indices were ±3 and for kurtosis indices were ±10 [38].

Two types of reliability were obtained for the WHO-5 Malay: internal consistency and test–retest reliability. Cronbach alpha values were used to establish internal consistency reliability. Cronbach alpha values of 0.70 were regarded as acceptable and 0.90 as excellent [39]. Pearson’s *r* correlation test was performed to establish 5-week test–retest reliability. The WHO-5 Malay scores at the initial assessment and at 5-week follow-up were subjected to the Pearson’s *r* correlation test.

Three types of validity were obtained for the WHO-5 Malay: factorial validity, concurrent validity, and convergent validity. First, factorial validity assess the extent to which a structure of a measurement is unidimensional or multidimensional [40]. Confirmatory factorial analysis (CFA) was performed to establish the factorial validity of the WHO-5. CFA is sensitive to sample size [41]. It appears that sample size needs to be adequate to ensure accurate estimation and construct interpretation. To counteract the issue of small sample size, we performed bootstrapping procedures (a bootstrap sample = 2000) with the Monte Carlo simulation and a 95% confidence interval [41]. Multiple fit indices such as chi-square (χ*^2^*), χ*^2^* to degree of freedom ratio, goodness of fit index (GFI), normed fit index (NFI), root mean square error of approximation (RMSEA), Tucker-Lewis fit index (TLI) and comparative fit index (CFI) were used to evaluate model fit. Recommended cut off values for these good fit indices are presented in Table 2 [42].

Second, concurrent validity, which is one of the two types of criterion validity, establishes the extent a measurement is comparable to an independent standard [43]. The WHO-5 is initially designed to assess mental health issues (i.e., depression and distress), hence previous studies have examined the concurrent validity of the scale by correlating it with the PHQ-9 [28] and the PAID-20 [26]. Following this line of inquiry, we sought to establish the concurrent validity of the WHO-5 Malay by correlating the scale with the Malay versions of the PHQ-9 and the PAID-20 using a series of Pearson’s *r* correlation tests.

Third, convergent validity, which is one of the two types of construct validity, establishes whether a measurement correlates with another similar constructs [44]. Good well-being has been shown to be correlated with greater self-efficacy [14,45] and better engagement in diabetes self-care behaviors [30,46,47]. Thus, we sought to establish the convergent validity of the WHO-5 Malay by correlating the scale with the Malay versions of the DMSES and the SDSCA in a series of Pearson’s *r* correlation tests.

## 3. Results

Table 1 presents descriptive statistics for study measures. Assumption of normality for all study measures was met with absolute values of the skewness (<3) and kurtosis (<10) indices found to be within the acceptable range.

### 3.1. Internal Consistency and Test–Retest Reliability

Cronbach’s alpha was 0.91 for the WHO-5 Malay. There was a significant correlation between the WHO-5 Malay scores at the initial assessment and the WHO-5 Malay scores at the 5-week follow-up (*r* = 0.69, *p* < 0.01). Taken together, internal consistency and test–retest reliability evidence for the WHO-5 Malay was established in the present study.

### 3.2. Factorial Validity

**Figure 1:**
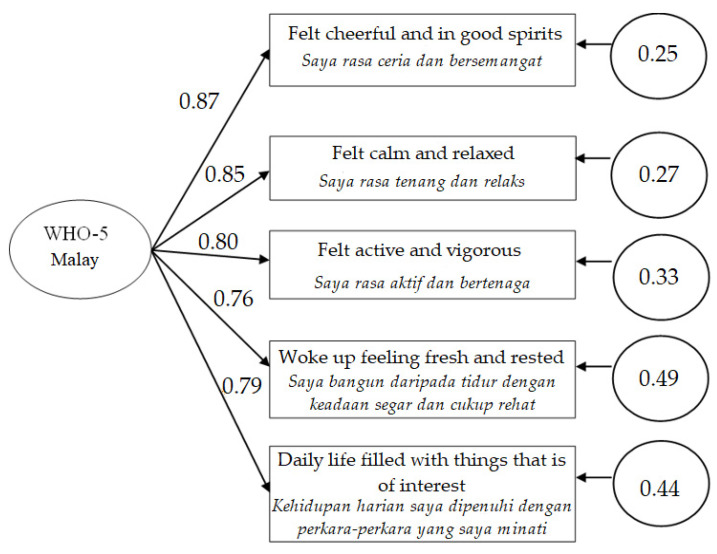
A unidimensional structure model of the Malay version of the WHO-5 Well-Being Index. Note. WHO-5 Malay = The Malay version of the WHO-5 Well-Being Index.

A CFA of a unidimensional structure model of the WHO-5 Malay is presented in Figure 1. We performed CFA using bootstrapping Monte Carlo procedure to examine the factorial validity of the WHO-5 Malay. Fitting the one-factor WHO-5 Malay model to the present sample yielded a good fit to the data with a chi-square value of χ*^2^*(5, *N =* 127) = 11.91, *p* = 0.04, with a ratio of χ*^2^ to df* = 2.38., GFI = 0.96, NFI = 0.97, RMSEA = 0.03, TLI = 0.97, and CFI = 0.98 (see Table 2). All values were considered a good fit and no further post hoc modifications were deemed necessary. All items loaded significantly on the WHO-5 construct with factors loadings ranging from 0.76 (Item 4) to 0.87 (Item 1). As expected, the factorial validity of the WHO-5 was found to be unidimensional. 

### 3.3. Concurrent Validity and Convergent Validity

As expected, there were significant negative correlations between the WHO-5 Malay and the Malay version of the PHQ-9 (*r* = −0.57, *p* < 0.01) and between the WHO-5 Malay and the Malay version of the PAID-20 (*r* = −0.40, *p* < 0.01). Additionally, as expected, there were significant positive correlations between the WHO-5 Malay and the Malay version of the DMSES (*r* = 0.51, *p* < 0.01) and between the WHO-5 Malay and the Malay version of the SDSCA (*r* = 0.27, *p* < 0.01). Both concurrent and convergent validity evidence for the WHO-5 Malay were established.

## 4. Discussion

Here, we reported the psychometric properties (reliability and validity) of the Malay version of the WHO-5 Malay in a sample of T2DM patients within the primary healthcare setting. As found in diabetes patients in an Australian community (α = 0.90; [28]) and in Dutch diabetes patients secondary care hospitals (α = 0.93; [26]), an excellent Cronbach alpha was obtained for the WHO-5 Malay in the present study. Despite different language versions, the Cronbach alpha obtained in the present sample was relatively higher than Thai (α = 0.87; [48]), Turkish (α = 0.83; [49]), and Japanese diabetes patient samples (α = 0.89; [50]). Participants’ scores of the WHO-5 Malay at the initial assessment were found to be significantly correlated with their scores of the same scale at 5-week follow-up. To the best of our knowledge, this study is the first to report the 5-week test–retest reliability of the WHO-5.

In terms of factorial validity, as with previous studies, our CFA findings lend support for a unidimensional structure of psychological well-being in the present sample [26]. Evidence for the concurrent and convergent validity of the WHO-5 Malay was established in the present study. As for concurrent validity, the WHO-5 Malay was found to be negatively correlated with depression and diabetes-related distress, lending support to the WHO-5 Malay as a valid screening tool for depression [23] and diabetes-related distress [26]. As far as the study population is concerned, our study was in agreement with Hajos et al. (2012). In particular, Hajos et al. (2012) found moderate to strong correlation between the WHO-5 and the measures of the PHQ-9 and the PAID-20 [26] in a sample involving Types 1 and 2 diabetes patients in the Netherland. However, cross-cultural validation is needed to ensure that the WHO-5 functions as intended and shares similar properties as the original in other language versions. Nonetheless, our present study suggests that the WHO-5 Malay is suitable within the busy primary healthcare clinics for screening emotional distress as part of a routine review [51]. As for convergent validity, consistent with existing diabetes literature, the WHO-5 Malay was found to be correlated with diabetes management self-efficacy and diabetes self-care behaviors, lending further support to the notion that individuals’ well-being would correlate with diabetes management self-efficacy and engagement to practicing diabetes self-care behaviors [52]. These findings represent important indications towards the achievement of optimal diabetes management and clinical outcomes [53,54]. In terms of clinical values, the WHO-5 Malay represents a valuable monitoring tool for clinical review, providing greater insight towards a more effective and holistic management that meet the needs of patients in local settings [54].

The present study is not without limitations. The present sample was limited to a clinical population of Type 2 diabetes patients, raising the issue of generalizability. The present sample size was considered small for confirmatory factor analysis [55]. Future research can extend the use of the WHO-5 Malay in other clinical populations with a larger sample size. The predictive validity of the WHO-5 Malay was not assessed in the present study. The validity of the scale can be further investigated by examining its relationships with diabetes complications, mortality, and morbidity rates with a longitudinal study design.

## 5. Conclusions

The objective of the present study was to assess the reliability and validity of the WHO-5 Malay in T2DM patients in a primary care setting. This study found that the translated version of the scale has promising psychometric properties and represents a promising tool that informs healthcare providers in making effective and holistic diabetes management.

## Figures and Tables

**Table 1:** Descriptive statistics for study measures.

Measures	M	SD	Skewness	Kurtosis
WHO-5 Malay	68.60	17.52	−0.19	−0.26
PHQ-9	4.44	4.39	2.30	7.39
PAID-20	26.81	16.61	0.25	−0.79
DMSES	142.40	28.61	0.00	−0.58
SDSCA	3.27	1.20	0.14	−0.39

Note: WHO-5 Malay = The Malay version of the WHO-5 Well-Being Index, PHQ-9 = Patient Health Questionnaire, PAID-20 = Problem Areas in Diabetes, DMSES = Diabetes Management Self-Efficacy Scale, SDSCA = Summary of Diabetes Self-Care Activities Measure.

**Table 2:** Model fit indices of the WHO-5 Malay and their recommended cut-off values.

Fit Indices	WHO-5 Malay Well-Being Index	Cutoff Values ^†^
Ratio of χ*^2^ to df*	2.38	≤2 or 3
Goodness of Fit Index	0.96	≥0.95
Normed Fit Index	0.97	≥0.95
Root Mean Square Error of Approximation	0.03	<0.06
Tucker–Lewis fit Index	0.97	≥0.95
Comparative fit Index	0.98	≥0.95

^†^ Schreiber et al. (2006).

## Data Availability

The data presented in study are available on request from the corresponding authors. The data are not available publicly due to privacy or ethical restrictions.
